# Design and validation of a new machine-learning-based diagnostic tool for the differentiation of dermatoscopic skin cancer images

**DOI:** 10.1371/journal.pone.0284437

**Published:** 2023-04-14

**Authors:** Amin Tajerian, Mohsen Kazemian, Mohammad Tajerian, Ava Akhavan Malayeri

**Affiliations:** 1 School of Medicine, Arak University of Medical Sciences, Arak, Iran; 2 School of Mechanical Engineering, College of Engineering, University of Tehran, Tehran, Iran; University of Wisconsin-Eau Claire, UNITED STATES

## Abstract

**Background:**

Skin cancer is the most common cancer in the United States. Current estimates are that one in five Americans will develop skin cancer in their lifetime. A skin cancer diagnosis is challenging for dermatologists requiring a biopsy from the lesion and histopathological examinations. In this article, we used the HAM10000 dataset to develop a web application that classifies skin cancer lesions.

**Method:**

This article presents a methodological approach that utilizes dermoscopy images from the HAM10000 dataset, a collection of 10015 dermatoscopic images collected over 20 years from two different sites, to improve the diagnosis of pigmented skin lesions. The study design involves image pre-processing, which includes labelling, resizing, and data augmentation techniques to increase the instances of the dataset. Transfer learning, a machine learning technique, was used to create a model architecture that includes EfficientNET-B1, a variant of the baseline model EfficientNET-B0, with a global average pooling 2D layer and a softmax layer with 7 nodes added on top. The results of the study offer a promising method for dermatologists to improve their diagnosis of pigmented skin lesions.

**Results:**

The model performs best in detecting melanocytic nevi lesions with an F1 score of 0.93. The F1 score for Actinic Keratosis, Basal Cell Carcinoma, Benign Keratosis, Dermatofibroma, Melanoma, and Vascular lesions was consecutively 0.63, 0.72, 0.70, 0.54, 0.58, and 0.80.

**Conclusions:**

We classified seven distinct skin lesions in the HAM10000 dataset with an EfficientNet model reaching an accuracy of 84.3%, which provides a promising outlook for further development of more accurate models.

## Introduction

Cancer is one of the significant healthcare burdens across the world. Global statistics suggest that almost 10.0 million deaths (9.9 million excluding non-melanoma skin cancer) were due to cancer in 2020 [1]. Skin cancer is the most common cancer in the United States. Current estimates are that one in five Americans will develop skin cancer in their lifetime [2, 3]. Fair-skinned people are more sun-sensitive than dark-skinned as they have adequate pigment protection; therefore, when exposed to UV radiation, the rate of being affected is higher in the fair-skinned population [4].

Several layers make a normal skin tissue, including the epidermis, dermis, and subcutaneous layer [5]. In the short term, within 24 hours of exposure to UV radiation, there is a noticeable increase in extracellular LDH membrane leakage and upregulation of inflammatory cytokines such as IL-1β in an in vitro environment [6]. Over the long term, unprotected exposure to UV radiation damages the DNA of skin cells and produces genetic defects or mutations [7]. These mutations can cause skin cancer in cells like melanocytes and epidermal keratinocytes [8], which is a growing global health concern due to its increasing incidence and mortality rates, particularly among seniors. Skin cancer is broadly categorized into melanoma and non-melanoma types [9].

The two main subgroups of non-melanomatous skin cancer are BCC and SCC, the most common skin cancer types and account for about 80% and 20% of skin neoplasms, respectively. On the other hand, melanoma is only responsible for about 2% of skin malignancies. However, it concerns physicians since it is the most lethal form of skin cancer [10]. Melanoma development is multifactorial, stemming from an interaction between genetic susceptibility and environmental exposures. Prevention strategies involve modifying the environmental risk factors and identifying individuals with phenotypic risk factors for increased follow-ups [11]. Doctors typically order a biopsy to detect skin cancer. Whereas a histopathologic study is usually required, early diagnosis can make a tremendous difference in curing the disease [12].

The HAM10000 dataset is a collection of 10015 images of pigmented skin lesions, categorized into seven subgroups:

1. Actinic Keratosis [AKIEC]: Non-invasive type of squamous cell carcinoma that can be treated locally without surgery.
2. Basal Cell Carcinoma [BCC]: A type of epithelial skin cancer that rarely spreads, but if left untreated, it might become aggressive and relapse.
3. Benign Keratosis [BKL]: "Benign keratosis" is a generic class that includes seborrheic keratoses, lichen-planus-like keratoses, and solar lentigo. The three subgroups may look different dermatoscopically, but they are grouped as they are similar biologically and often reported under the same generic term histopathologically.
4. Dermatofibroma [DF]: This skin lesion is either benign growth or an inflammatory response to minor trauma.
5. Melanoma [MEL]: Melanoma is a cancerous tumor that develops from melanocytes and can take many forms. It can be treated with a primary surgical procedure if caught early enough.
6. Melanocytic Nevi [NV]: Skin lesions are benign neoplasms of melanocytes and appear in various shapes and sizes. From a dermatoscopic standpoint, the variants may differ dramatically.
7. Vascular Lesions [VASC]: Cherry antifoams, angiokeratomas, and pyogenic granulomas are examples of benign or malignant angiomas [13–15].

This collection is used to create machine-learning algorithms that allocate every lesion to one of the predefined subgroups. A study was done in 2018 to compare the accuracy of the diagnosis of human readers and the State-of-the-art machine-learning algorithms. In this study, 511 human readers, including 283 (55.4%) board-certified dermatologists, 118 (23.1%) dermatology residents, and 83 (16.2%) general practitioners from 63 countries, were involved. State-of-the-art machine-learning classifiers outperformed human experts in diagnosing pigmented skin lesions with a mean of 2.01 more correct diagnoses in each 30-image batch and should have a more critical role in clinical practice [16].

The integration of artificial intelligence (AI) and machine learning in diagnosing diseases is garnering significant attention in the medical community. The advent of these technologies has led to a paradigm shift in the approach to diagnosing cancer, with the potential to improve diagnostic accuracy, reduce errors, and enhance patient outcomes [17, 18]. By leveraging vast amounts of data, machine learning algorithms can identify patterns and features in skin lesions that may not be discernible to the human eye. This can aid physicians in making more informed decisions, providing precise and personalized treatment options, and ultimately improving patient care.

Over the past few years, researchers have been exploring the use of artificial intelligence (AI) and machine learning (ML) to diagnose skin cancer. The technology has shown great potential in identifying skin lesions that may be indicative of skin cancer.

One of the prominent studies on this topic was conducted by Esteva et al. (2017) at Stanford University, where a deep learning algorithm was trained to identify skin cancer from images. The researchers used a dataset of over 129,000 images of skin lesions and achieved a classification with AUC of 0.91. This is a promising result, as it suggests that AI could be a valuable tool for dermatologists in diagnosing skin cancer [19]. With the proliferation of smartphone users, Esteva et al. proposed that we could potentially offer affordable and accessible diagnostic care to everyone by 2021. Regrettably, this vision did not come to fruition. Nevertheless, in pursuit of this laudable objective, we embarked on the creation of a pioneering application, which serves as our inaugural stride toward its realization.

In this article, we present a deep learning model for the classification of dermatoscopic skin lesions images. The model was trained on the HAM10000 dataset, which consists of 10,015 dermatoscopic images of skin lesions, and achieved an accuracy of 84.3% on the test set. The study used transfer learning and fine-tuning techniques to train an EfficientNetB1 model for the classification task. The results show that the model can assist dermatologists in the diagnosis of skin lesions, potentially improving the accuracy and speed of diagnosis. The trained model can be used as a tool to assist dermatologists in their clinical practice, and future work may involve integrating the model into a user-friendly application or system.

## Methods

### Study population

Dermatoscopy, also known as dermoscopy, epiluminescence microscopy, or skin surface microscopy, is a non-invasive, in-vivo method traditionally applied to evaluate suspicious skin lesions [20]. This well-used method improves the diagnosis of benign and malignant pigmented skin lesions compared to examination with the unaided eye [21]. The HAM10000 dataset is a set of 10015 dermatoscopic images collected over 20 years from two different sites, the Department of Dermatology at the Medical University of Vienna, Austria, and the skin cancer practice of Cliff Rosendahl in Queensland, Australia [14]. All data records of the HAM10000 dataset are deposited at the Harvard Dataverse. These Images and metadata are also accessible at the public ISIC archive through the archive gallery and standardized API calls (https://isic-archive.com/api/v1) [14]. The majority of lesions have been confirmed by pathology. At the same time, the ground truth for the rest of the cases was either follow-up, expert consensus, or confirmation by in-vivo confocal microscopy [14].

### Ethics concerning human participants

The HAM10000 dataset used in this study was initially extracted from the office of the skin cancer practice of Cliff Rosendahl (CR, School of Medicine, University of Queensland). The data from this database was extracted after institutional ethics board approval (University of Queensland, Protocol-No. 2017001223) [14]. As we did not directly deal with human subjects and used anonymized and unidentifiable data from previously published and openly accessible studies, we declare that the ethical concerns do not apply to this study.

### Statistical analysis

Descriptive statistics were used to summarize the dataset’s characteristics and were presented as frequencies, percentages for categorical variables, mean ± standard deviation, and median (min-max) for continuous variables. The chi-square test was used to compare the categorical variables. The independent-sample T-test was used to compare means. Pearson correlation coefficients and linear regression were used to compare continuous variables. A P-value of less than 0.05 was considered to be statistically significant. Statistical analysis was conducted using IBM SPSS statistics 26 (IBM Corp., Armonk, NY, USA) to analyze the data. Plotly, Seaborn, and Matplotlib were used to plot data which are open-source libraries for python.

### Study design and workflow

#### Image pre-processing

The data was obtained from Kaggle, available via a CC0: Public Domain License. It is appropriately anonymized and does not contain any identifiable features of the participants. As the dataset images were not labelled and were out of order, each image was first labelled using the dataset’s metadata by transferring them into its respective folder. Then it was randomly split into a training set containing 8015 (80%) cases and a test set containing 2000 (20%) images.

The images were resized to (240, 240, 3) tensors, as the EfficientNetB1 architecture, which was used to build the model, has the optimum performance with this size [22]. The well-known bilinear interpolation resampling technique was used in image processing to resize the images.

**Fig 1:**
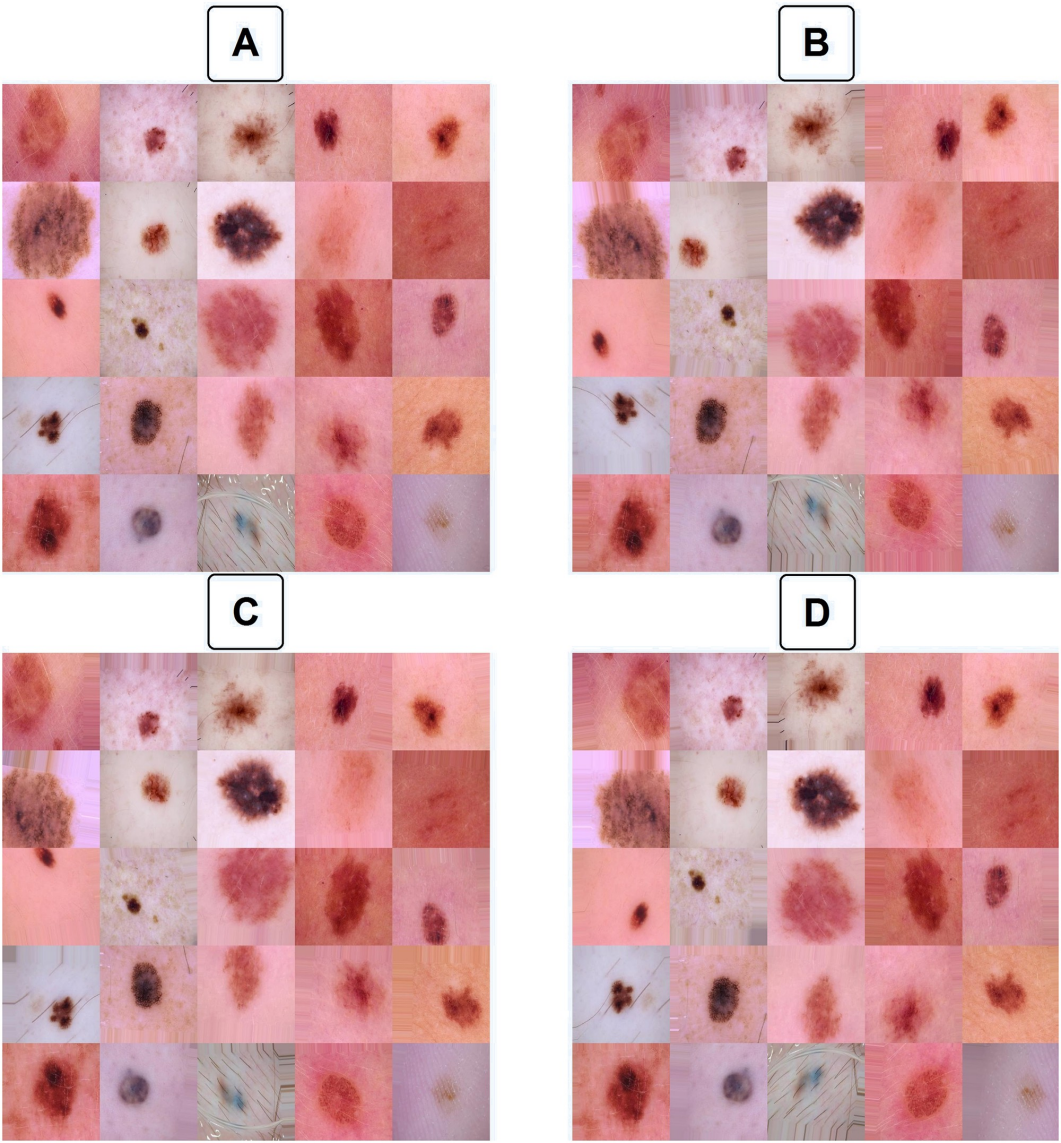
Data augmentation. Illustration of how data augmentation algorithm works. A, 25 intact random images from the dataset; B, C, and D, random data augmentations applied to that same 25 images, which would then feed to the model.

In order to artificially increase the instances, a data augmentation technique is used to generate new sample images. This technique consisted of a random width shift from -20% to +20% of image width and a random height shift from -20% to +20% of image height, and a random max 0.2-degree shear angle in a counter-clockwise direction to rectify the perception angle and also random horizontal and vertical flip was applied. Empty pixels were then filled by the nearest pixel. To better understand this procedure, we randomly applied data augmentation to 25 images 3 times, and the results are shown in Fig 1. Also, S1 File in supplementary materials illustrates 600 frames of random augmentations for 9 lesions with a frame rate of 10 FPS.

#### Model architecture for transfer learning

It is generally not a good idea to train a very large Deep Neural Network from scratch as training such large models with at least 200 to 300 hidden layers requires a massive amount of resources and time not everyone has, instead using existing pre-trained models that accomplishes a similar task is a much reasonable idea. Transfer learning is a research problem in machine learning that focuses on storing knowledge in the process of solving one problem and applying the gained accumulated knowledge to accelerate the learning in new different, but related tasks [23, 24]. Transfer learning nets are trained on large datasets, and the model parameters of each layer could be manually set to be frozen so that they will not change during retraining.

**Fig 2:**
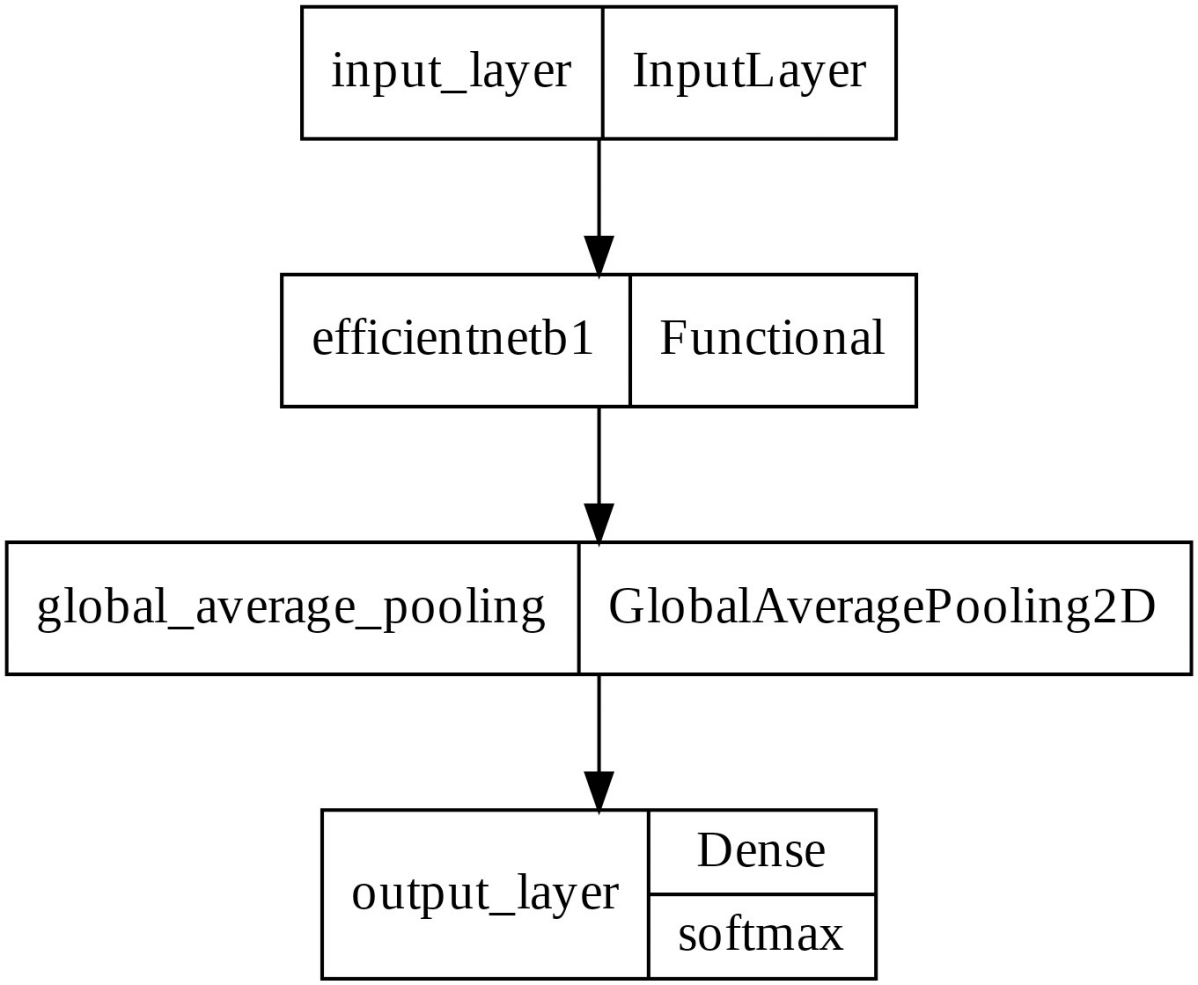
The model’s architecture. The 340 layers of the EfficientNET-B1 is collapsed.

The Efficient nets are a family of neural networks with the baseline model constructed with the Neural Architecture Search technique. The EfficientNET-B1, a variant of the baseline model EfficientNET-B0 which is created through compound scaling, is the backbone of our model. We deleted the top layer of EfficientNET-B1, then a Global average pooling 2D layer and a softmax layer with 7 nodes added on top. The model architecture is shown in Fig 2. As the EfficientNET-B1 has 340 layers, it is not possible to show all layers, so an expanded version of the architecture is available in supplementary materials.

#### Feature extraction and fine-tuning

The top layer of the models has nodes equal to several classes and a softmax activation function which is used as the last activation function of a neural network to normalize the output of a network to a probability distribution over predicted output classes. In feature extraction transfer learning, this top layer gets replaced by a new softmax-activated layer with nodes equal to the new problem (7 nodes in this particular image classification problem). Then all other pre-trained model layers are set frozen so that only this new layer’s parameters get trained to adjust the outputs to be more suited to a new problem.

**Fig 3:**
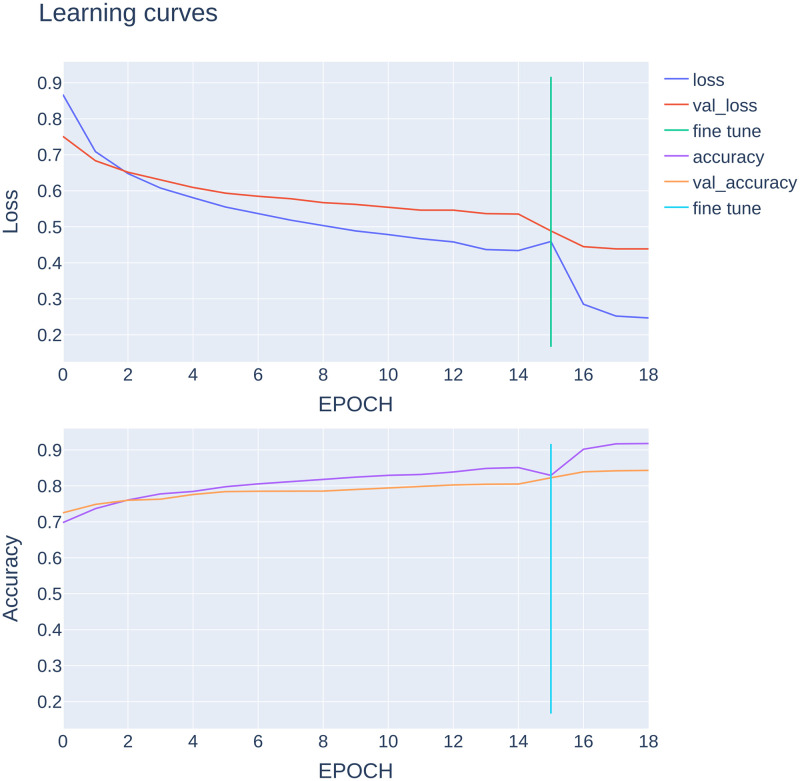
Learning curves. The alterations in loss and accuracy of the model’s predictions per epoch is illustrated.

At first, we freeze all the layers to train our new layers’ parameters for feature extraction; then, the model is trained for 15 epochs. The learning rate was set at 0.001 primarily, but after 12 epochs, as the learning curve reached a Plateau, the learning rate reduced to 0.0001 for the remaining epochs. After feature extraction, the top 50 layers of EfficientNET-B1 were unfrozen to fine-tune the EfficientNET-B1 itself for our specific image classification problem. The learning curve is illustrated in Fig 3. The model was fine-tuned for 4 more epochs starting with the learning rate of 1.0e-4 and decreasing to 10% for each epoch using the Learning Rate Scheduler callbacks. However, as the learning curve reached a plateau in the last epoch, the learning curve decreased even more, and it reached 1.0e-8 for the last epoch.

The Adam algorithm, a simple-to-use, computationally efficient, and effective method, was used to optimize the categorical cross-entropy loss function to the minimum amount. The model was built and trained using the TensorFlow library v2.11 on python. The hyperparameters of the algorithms were tuned to achieve the desired outcome.

## Results

### Dataset description and statistical analysis

**Fig 4:**
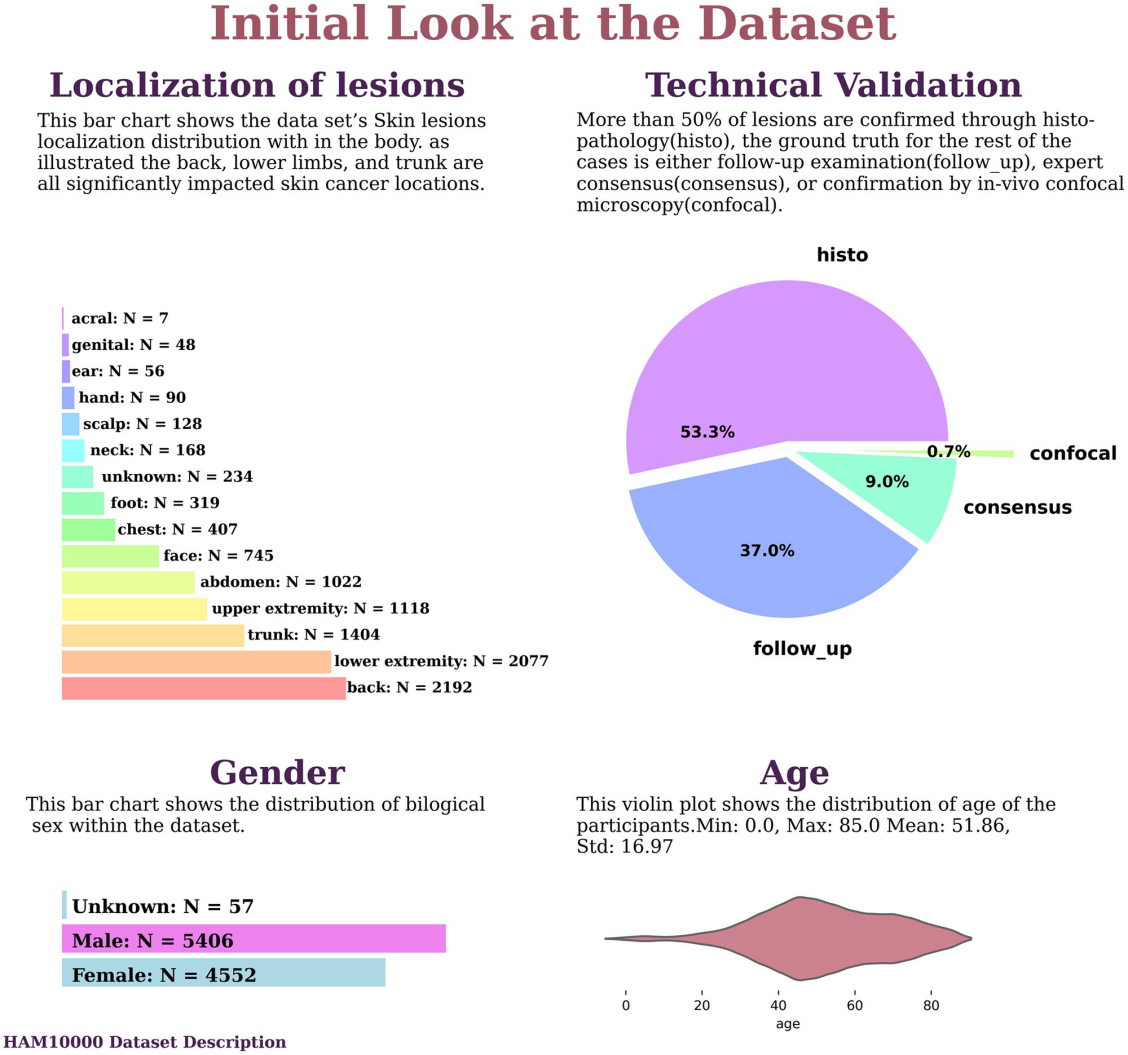
Dataset description.

This population contains information about 10015 participants with a mean age of 51.86±16.96 which was only reported for 9958 cases. The biological sex of the 5406 (54.1%) participants was Male, 4552 (45.5%) were Female, and for 57 (0.6%) of them, the gender was unknown. The skin lesion images were taken from various parts of the body. The back with 2192(21.9%) images, the lower extremities with 2077(20.7%) images, the trunk with 1404(14.0%) images, the upper extremities with 1118(11.2%) images, and the abdomen with 1022(10.2) images were the 5 most involved parts of the body; other ports prevalence is described in Fig 4.

The diagnosis for each lesion was confirmed via a specific route; 5340 (53.3%) lesions were confirmed through histopathologic examinations, 3704(37.0%) lesions were confirmed by follow-up examinations, 902(9.0%) lesions were confirmed by expert consensus, and the ground truth for 69(0.7%) of lesions was confirmation by in-vivo confocal microscopy Fig 4.

**Fig 5:**
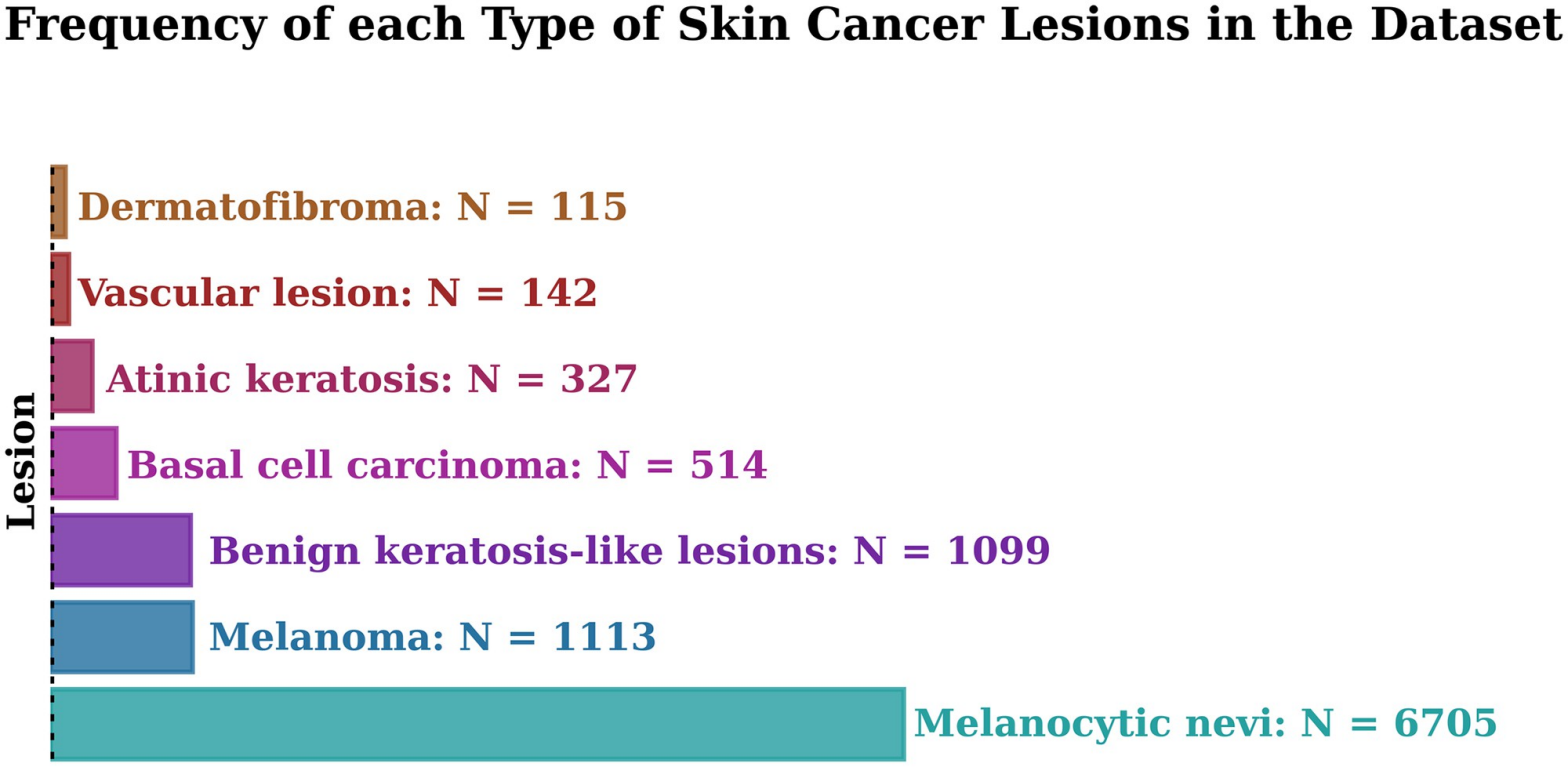
Frequency of each type of skin cancer lesions in the dataset.

This dataset includes a representative collection of all essential diagnostic categories in pigmented lesions, but these categories were highly imbalanced, as illustrated in Fig 5. Most lesions were melanocytic nevi with 6705(66.9%) images. The rest of the lesions were melanoma (N = 1113, 11.1%), benign keratosis (N = 1099, 11.1%), basal cell carcinomas (N = 514, 5.1%), actinic keratosis (N = 327, 3.3%), vascular skin lesions (142, 1.4%), and dermatofibroma (115, 1.1%).

**Fig 6:**
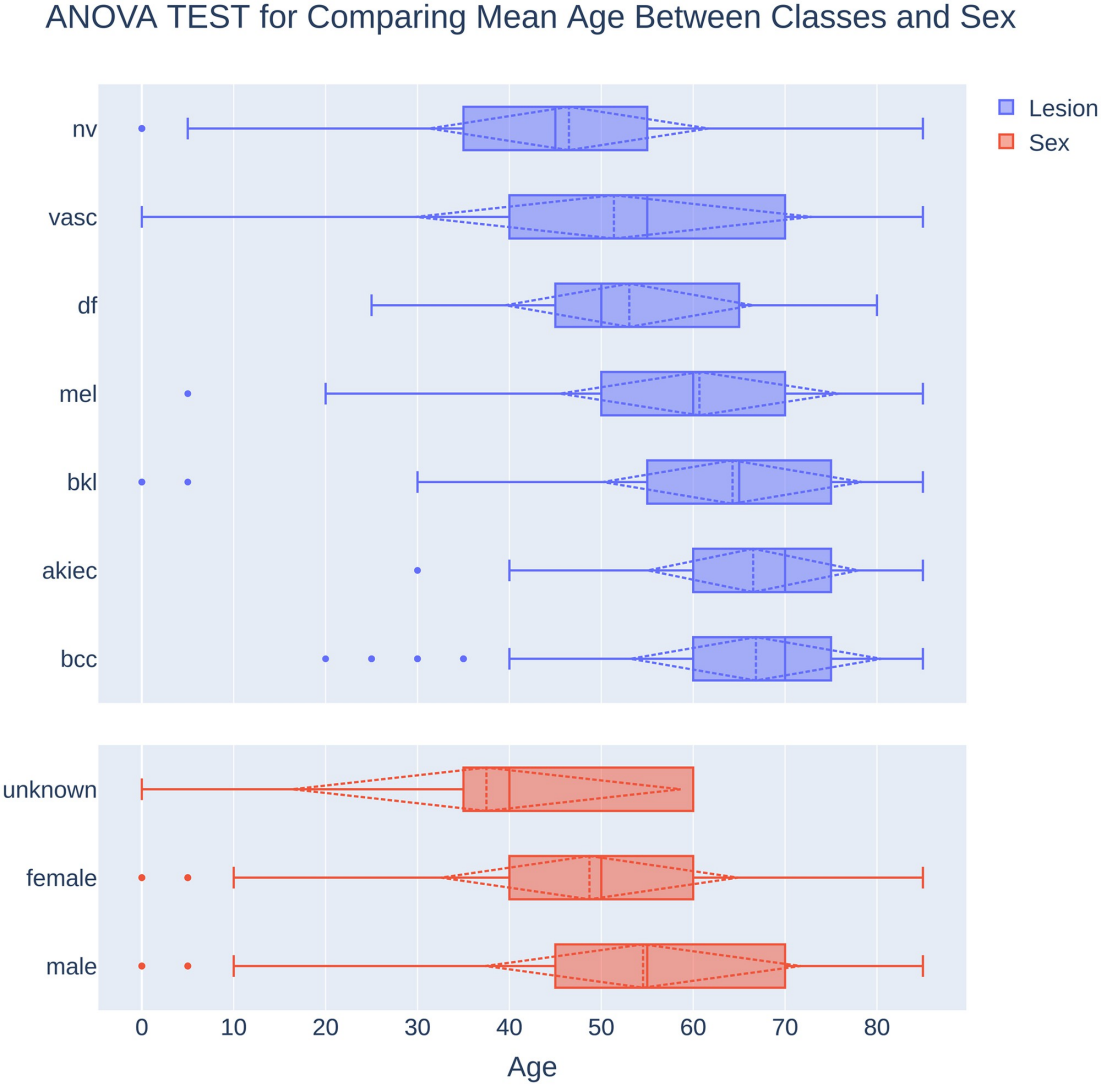
ANOVA test to compare mean age between different classes of skin cancer and gender. Dashed lines indicate Mean and ± one standard deviation interval from Mean. Dots indicate outliners.

To establish whether there is a meaningful relationship between age and the type of skin cancer, we conducted an ANOVA test to compare mean age between different classes of skin cancer. A one-way ANOVA demonstrated that there is a statistically significant main effect of skin cancer type on age (F [6, 9951] = 470.117, p < .001); see Fig 6. The mean age and standard deviation for each skin cancer type are described in Table 1. Post hoc analyses using the Scheffé post hoc criterion for significance indicated that the average age was significantly lower in the melanocytic nevi class than in all the other 6 skin cancer classes with p values less than 0.001. the results of the Post hoc analyses are available in supplementary materials.

**Table 1:** Comparing the mean age between skin cancer lesions.

Lesion	N	Mean	Std. Deviation	Std. Error
akiec	327	66.529	11.4762	.6346
bcc	514	66.829	13.6570	.6024
bkl	1089	64.284	14.1207	.4279
df	115	53.043	13.5513	1.2637
mel	1111	60.680	15.1898	.4557
nv	6660	46.477	15.1833	.1860
vasc	142	51.373	21.6447	1.8164

AKIEC: Actinic Keratosis; BCC: Basal Cell Carcinoma; BKL: Benign Keratosis; DF: Dermatofibroma; MEL: Melanoma; NV: Melanocytic Nevi; VASC: Vascular Lesions.

Also, to compare age between different classes of sex, a one-way ANOVA was conducted, which demonstrated that the 5400 male participants with an average age of 54.54±17.15, the 4548 female participants with an average age of 48.71±16.15; and the 10 participants with unknown sex had an average age of 37.50±22.26. The effect of gender, therefore, was significant (F [2,9955] = 8.76, p < 0.001) in the age of the people with skin cancer in this dataset (see Fig 6).

### ML model performance validation

The Machine Learning model was trained and tested on a Google Colaboratory environment with an Intel(R) Xeon(R) 2.30GHz CPU processor and 13GB of RAM and NVIDIA Tesla T4 CUDA enabled GPU processor with CUDA 11.2 which has designed for high-performance computing, deep learning training and inference, machine learning, and data analytics. The model was created with Python 3.8.6, TensorFlow 2.11, Scikit-Learn 1.0.2, and Numpy as dependencies.

This section presents the experimental results of our model trained on the HAM10000 dataset. The model was trained for 19 epochs with a batch size of 32, and in every epoch, training accuracy, training loss, and validation accuracy, validation loss was calculated. We used an Adaptive Momentum (Adam) optimizer on Categorical Cross Entropy loss function with a dynamic learning rate (LR) starting from 0.001. For fine-tuning in order to make the optimizer converge faster and get closer to the global minimum of the loss function, the learning was set high in early epochs, and by getting closer to the global optimum, the learning rates decreased to take tiny steps toward the global optimum. Also, we used the ReduceLROnPlateau callback to reduce the LR even more if the validation loss did not improve after 3 epochs. The metrics and LR for each epoch are described in Table 2.

**Table 2:** The model alterations description per epoch during training.

epoch	loss	accuracy	val_loss	val_accuracy	LR ~
0	0.8674	0.6979	0.7513	0.7250	1.0e-03
1	0.7089	0.7367	0.6836	0.7485	1.0e-03
2	0.6479	0.7608	0.6517	0.7600	1.0e-03
3	0.6077	0.7777	0.6297	0.7630	1.0e-03
4	0.5808	0.7844	0.6094	0.7760	1.0e-03
5	0.5550	0.7976	0.5935	0.7840	1.0e-03
6	0.5368	0.8055	0.5849	0.7850	1.0e-03
7	0.5186	0.8125	0.5781	0.7860	1.0e-03
8	0.5035	0.8180	0.5672	0.7855	1.0e-03
9	0.4886	0.8243	0.5623	0.7900	1.0e-03
10	0.4785	0.8292	0.5548	0.7940	1.0e-03
11	0.4668	0.8316	0.5463	0.7995	1.0e-03
12	0.4581	0.8386	0.5463	0.8025	1.0e-03
13	0.4370	0.8485	0.5367	0.8045	1.0e-04
14	0.4341	0.8509	0.5355	0.8050	1.0e-04
15	0.4593	0.8292	0.4886	0.8225	1.0e-04
16	0.2851	0.9018	0.4450	0.8390	1.0e-05
17	0.2522	0.9169	0.4387	0.8420	1.0e-06
18	0.2470	0.9177	0.4387	0.8430	1.0e-08

loss: training set loss; val_accuracy: training set accuracy; val_loss: test set loss; val_accuracy: test set accuracy; LR: learning rate.

**Fig 7:**
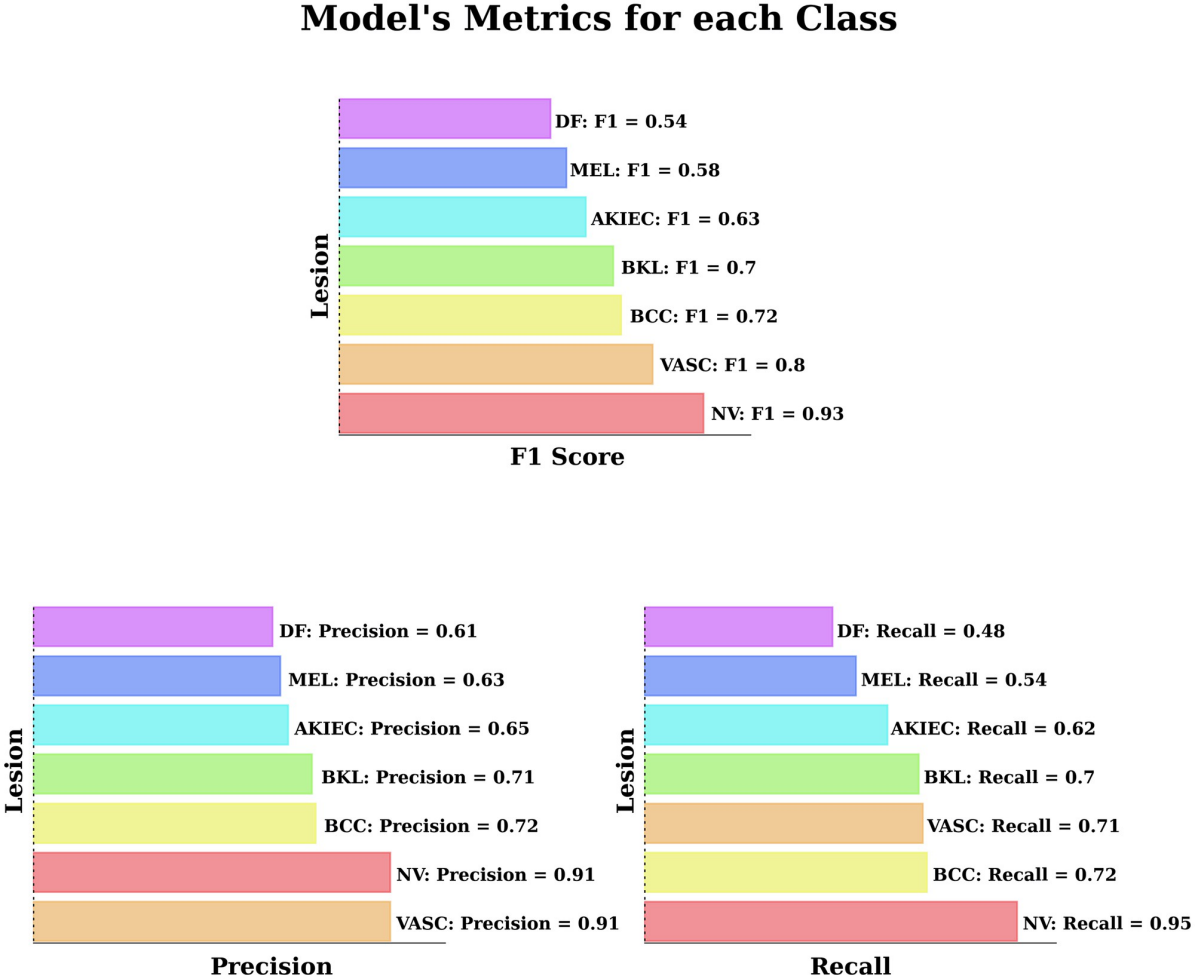
Model metrics for each class. AKIEC: Actinic Keratosis; BCC: Basal Cell Carcinoma; BKL: Benign Keratosis; DF: Dermatofibroma; MEL: Melanoma; NV: Melanocytic Nevi; VASC: Vascular Lesions.

The model’s precision and Recall and F1 Scores are described and compared in Fig 7 based on each class. As demonstrated in this Figure, the model performs best in detecting melanocytic nevi lesions with an F1 score of 0.93. This performance difference between the classes is mainly due to the highly imbalanced classes of the dataset. As the model gets trained with lots of melanocytic nevi images (about 5364 melanocytic nevi images compared to 92 dermatofibroma images), inevitably, the model learns more patterns to detect this specific class and higher performance in this class. As demonstrated in Table 2, the final model’s accuracy on unseen images of the test set was 84.30%, with a loss of 0.4387.

### The model’s worst predictions

**Fig 8:**
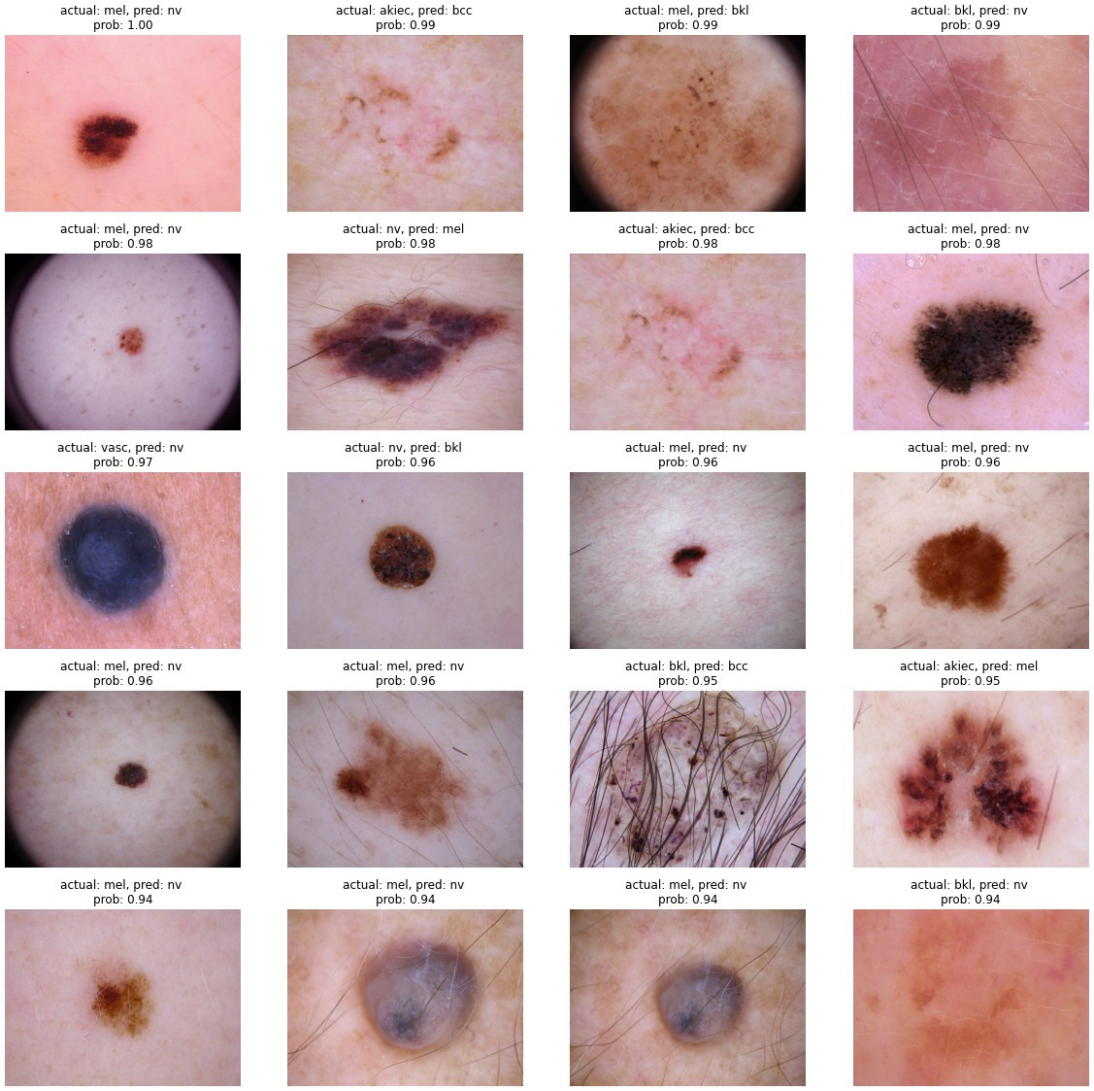
The top 20 most wrong predictions. Actual: the true label of each image; pred: the model’s predicted class; prob: the probability inferred to the predicted class by the model.

After making the predictions for all test images, we sorted the wrong predictions by their inferred probability to find those images that the model guessed wrong with the highest confidence. This procedure helps find both the dataset and the model’s problems. It is possible that an image is incorrectly labelled and the model is actually doing right; for instance, in the top 20 most wrong predictions (Fig 8), we found 2 identical images, and one of them should be deleted from the dataset.

### Web application

We also developed a web application to enable researchers to assess our model by uploading their malignant skin lesion images to the application and getting the results instantly.

Please note that this application has no clinical or diagnostic value and is for research purposes only. Some randomized clinical trials should be conducted to find clinical evidence supporting the accuracy of these models. Our application is available online at https://tajerian.info/ham10000. This application is also available offline and installable on computer devices on both windows and Linux, which can be downloaded from GitHub (https://github.com/tajerian/ham10000-app).

## Discussion

In recent years, artificial intelligence (AI) and machine learning (ML) have shown great potential in diagnosing skin cancer. The integration of these technologies in healthcare can improve diagnostic accuracy, reduce errors, and enhance patient outcomes. Skin cancer is a significant healthcare burden, with increasing incidence and mortality rates globally. Melanoma and non-melanoma types are the most common types of skin cancer. The HAM10000 dataset is a collection of 10015 images of pigmented skin lesions, categorized into seven subgroups. The dataset is widely used in the field of dermatology for the training and evaluation of machine learning models for skin cancer diagnosis.

### State of the art

One of the pioneering studies on this topic was conducted by Esteva et al. (2017) at Stanford University, where a deep learning algorithm was trained to identify skin cancer from images. The researchers used a dataset of over 129,000 images of skin lesions. This algorithm achieved a classification accuracy of 72.1% for the model with 3 classes and accuracy of 55.4% for the model with 9 classes, outperforming a group of 21 board-certified dermatologists in identifying melanoma. However, this study did not use a dermatoscopic imaging modality. This study showed that AI could be a valuable tool for dermatologists in diagnosing skin cancer [19].

Tschandl et al. (2018) conducted an impressive study in which they developed a CNN-based classification model. The model was trained on a large dataset of 7895 dermoscopic and 5829 close-up images of lesions that were excised at a primary skin cancer clinic. This combined CNN (cCNN) model was tested on a set of 2072 unknown cases and compared with results from 95 human readers who were medical personnel, including 62 board-certified dermatologists, with different experience in dermoscopy. This cCNN achieved a higher percentage of correct specific diagnoses compared with human raters but not compared with experts. Overall, this study provides compelling evidence for the potential of AI-based models in the field of dermatology [25].

Haenssle et al. (2018) conducted a ground-breaking study in the field of skin cancer detection, in which they sought to investigate whether a deep learning convolutional neural network (CNN) could accurately diagnose skin lesions, outperforming human dermatologists. This study was the first to compare the diagnostic performance of a CNN with a large international group of 58 dermatologists, including 30 experts. The results of the study were remarkable, as most dermatologists were outperformed by the CNN in identifying skin lesions. In this study Google’s Inception v4 CNN architecture was trained on a set of more than 100 000 digital images and corresponding disease labels [26].

The study suggested that regardless of a physician’s level of experience, they may benefit from the assistance of a CNN’s image classification when diagnosing skin lesions. This has important implications for the future of dermatology and suggests that AI-based models like CNNs could become an essential tool for dermatologists in the diagnosis and treatment of skin cancer [26].

Ahmadi Mehr et al. (2022) proposed a viable deep learning (DL) based method for the detection of skin cancer in lesion images. Using this method, they developed a system that can automatically detect skin lesions and classify them as malignant or benign. They used three databases containing clinical images of skin lesions to train and evaluate their system, which consisted of the Inception-ResNet-v2 convolutional neural network (CNN). The CNN was fine-tuned on their dataset to classify 16 different skin-disease conditions, including melanoma and non-melanoma skin cancers. The final product of the study is a trained CNN model that can classify skin lesions as malignant or benign with an accuracy of 94.5%±0.9%, which has the potential to assist dermatologists in early detection and treatment of skin cancer [27].

Trejic et al., in a paper published in 2021, concluded that family physicians have a 74.3% (95% confidence interval [CI], 56.7% to 87.5%) sensitivity in diagnosing malignant skin lesions [28]. Another study by Sellheyer and Bergfeld deduced that in comparison with histopathologic diagnosis, dermatologists correctly diagnosed skin lesions in up to 75% of cases, while this number for family physicians was 26% [29]. Alam et al., in their 2022 paper, compared their study with 12 other studies, all carried on the HAM10000 dataset and stated that the diagnostic accuracy was between 82.9% to 91% [30]. One study achieved an accuracy of 85.8% by selecting EfficientNet-B4 as the classification model [31]. We consummated the classification with an accuracy of 84.3% on the unseen images of the test data utilizing an EfficientNET-B1 model to classify the HAM10000 dataset.

### Weaknesses

One of the weaknesses of this work is the lack of diversity in the dataset. Although the dataset was large and diverse, it was still limited to a specific geographic region with fair skin. This means that the model may not generalize well to other populations with different skin types and genetic backgrounds. To address this, future work should focus on collecting and incorporating data from a wider range of populations. Another issue with this dataset is the imbalanced number of each class’s instances, which reduces the model’s ability to guess the classes with a lower number of instances correctly.

Another weakness of our work is the lack of validation on real patient data. Although the model performed well on the test dataset, it is important to validate its performance on real patient data to ensure that it is safe and effective to use in a clinical setting. This will require collaboration with medical professionals to collect and annotate real patient data, which can be time-consuming and challenging.

### Future directions

Although this study represents a significant contribution towards the early diagnosis of skin cancer, there are several directions that future research could take. For example, incorporating additional clinical features such as patient age, gender, and medical history could improve the accuracy of the model. Furthermore, exploring the use of other ML algorithms and techniques could improve the performance of the model even further.

## Conclusion

Despite all limitations discussed before, this study represents an important step forward in the development of automated tools for skin cancer diagnosis. Future studies could build on your work by expanding the dataset to include more diverse populations and additional diagnostic categories. Additionally, it would be interesting to explore the use of other imaging modalities in combination with ML models to further improve diagnostic accuracy.

The trained model can serve as a valuable tool to assist dermatologists in their clinical practice, potentially improving the accuracy and speed of diagnosis. Future work may involve integrating the model into a user-friendly application or system to make it more accessible to healthcare providers and patients.

## Supporting information

S1 File Data augmentation. Illustrates 600 frames of random augmentations for 9 lesions with a frame rate of 10 FPS.
(MP4)
